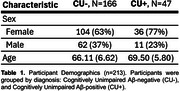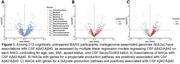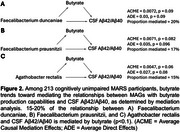# Fecal levels of short‐chain fatty acids and prominent bacterial taxa involved in their production are inversely associated with amyloid‐positive status

**DOI:** 10.1002/alz70856_098071

**Published:** 2025-12-24

**Authors:** Jessamine F Kuehn, Qijun Zhang, Margo B. Heston, Jea Woo Kang, Sandra Harding, Nancy J Davenport‐Sis, Robert L. Kerby, Emma C. Schiffmann, Joseph L. Wheeler, Eleanor Clements, Sushma Shankar, Alissa Mickol, Justus Zemberi, Hana Chow, Eric Zhang, Joseph Harpt, Aaliyah Mushtaque, Michelle Yoo, Aiden Cook, Cynthia M. Carlsson, Sterling C Johnson, Sanjay Asthana, Henrik Zetterberg, Kaj Blennow, Tyler K. Ulland, Barbara B. Bendlin, Federico E. Rey

**Affiliations:** ^1^ Department of Bacteriology, University of Wisconsin‐Madison, Madison, WI, USA; ^2^ University of California, San Francisco, Memory and Aging Center, San Francisco, CA, USA; ^3^ Wisconsin Alzheimer's Disease Research Center, University of Wisconsin School of Medicine and Public Health, Madison, WI, USA; ^4^ University of Wisconsin School of Medicine and Public Health, Madison, WI, USA; ^5^ Institute of Neuroscience and Physiology, Sahlgrenska Academy at the University of Gothenburg, Gothenburg, Sweden; ^6^ Department of Pathology and Laboratory Medicine, University of Wisconsin‐Madison, Madison, WI, USA; ^7^ Wisconsin Alzheimer's Disease Research Center, School of Medicine and Public Health, University of Wisconsin‐Madison, Madison, WI, USA

## Abstract

**Background:**

Short‐chain fatty acids (SCFA), including acetate, propionate, and butyrate, are abundant gut bacterial metabolites produced via the fermentation of dietary fibers and resistant starch. Several lines of evidence, particularly in preclinical mouse models, suggest a protective role of SCFA against Alzheimer's Disease (AD) pathology. In one study, supplementation of mice with tributyrin, a butyrate prodrug, significantly attenuated AD pathology. However, the relationships between SCFA, the bacterial taxa that produce them, and AD biomarkers require further elucidation in humans.

**Method:**

We assessed gut metagenomes and SCFA levels in fecal samples from 213 cognitively unimpaired Microbiome Alzheimer's Risk Study (MARS) participants (Table 1). The cohort was co‐enrolled in the Wisconsin Alzheimer's Disease Research Center and Wisconsin Registry for Alzheimer's Prevention, which track preclinical disease progression in middle‐aged and older adults at risk for AD. We sequenced DNA extracted from 213 fecal samples (one sample per participant, 30 million reads per sample), created metagenome‐assembled genomes (MAGs), and annotated their functions. We measured levels of the major SCFA in fecal samples using headspace gas chromatography. We performed multiple linear regressions between levels of cerebrospinal fluid (CSF) AD biomarkers and each SCFA or MAG, controlling for age, sex, body mass index, and *APOE* genotype.

**Result:**

We found an inverse association between amyloid positive status (CSF Aꞵ42/Aꞵ40 <0.046) and MAGs encoding propionate or butyrate production pathways. Fecal acetate, propionate, and butyrate levels were reduced in females and in participants with amyloid‐positive status. Mediation analysis detected a trend indicating that butyrate may mediate the inverse relationship between MAGs with butyrate production pathways and amyloid positive status.

**Conclusion:**

Relative abundances of MAGs encoding enzymes for propionate and butyrate production were reduced in amyloid‐positive participants in a cognitively unimpaired human cohort enriched for AD risk. These results, combined with the extensive literature in preclinical AD mouse models, suggest that SCFA may play a causal role in AD progression.